# Supplementary material for: Fabrication of an Extremely Cheap Poly(3,4-ethylenedioxythiophene) Modified Pencil Lead Electrode for Effective Hydroquinone Sensing
Source: Polymers (Basel). 2021 Jan 22;13(3):343. doi: 10.3390/polym13030343 (PMC7866211; doi:10.3390/polym13030343)
Supplement: Supplementary file 1 [file polymers-13-00343-s001.pdf]

Supplementary materials

Fabrication of an extremely cheap poly(3,4-ethylenedioxythiophene) modified pencil lead electrode for effective hydroquinone sensing

Jian-Yu Lu [1], Yu-Sheng Yu [2], Tung-Bo Chen [1], Chiung-Fen Chang [3],
Sigitas Tamulevičius [4], Donats Erts [5], Kevin C.-W. Wu 2 and Yesong Gu [1,*]

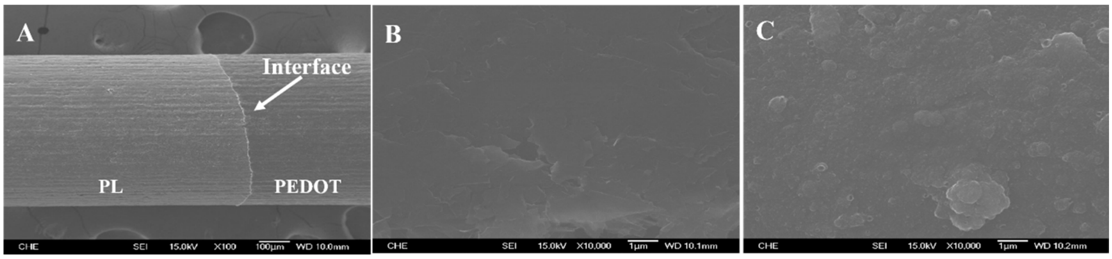

Figure S1. The surface images of PL and PEDOT/PL electrodes by SEM. (A) the interface of PEDOT film on the PL electrode. (B) and (C) were the enlarged images of PL and PEDOT film, respectively.

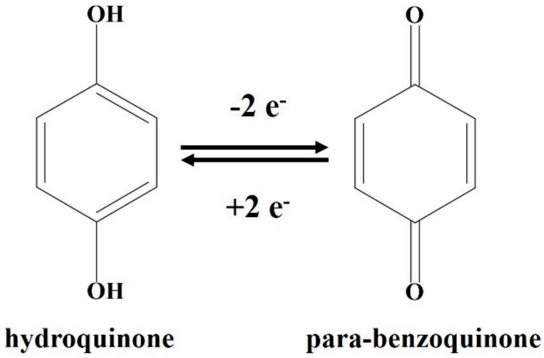

Figure S2. The oxidation and reduction mechanism for hydroquinone and para-benzoquinone

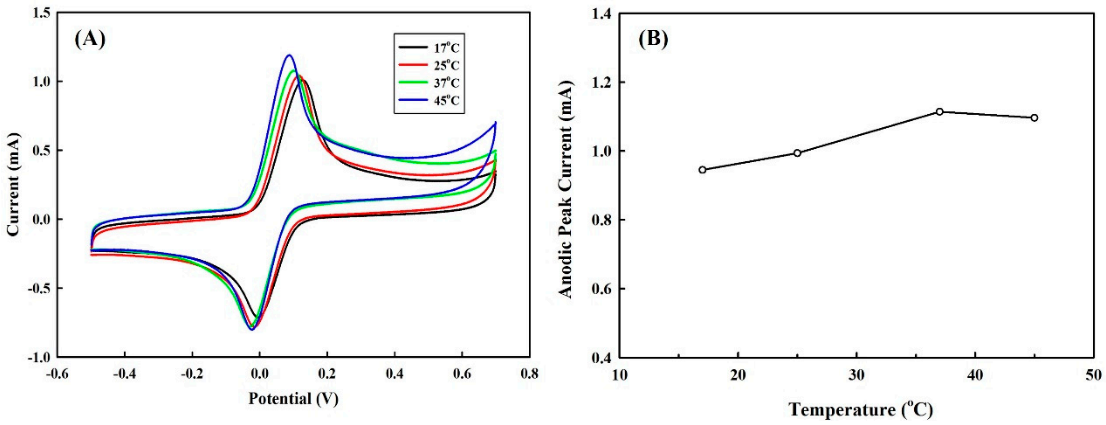

Figure S3. (A) The effect of temperature on the performance of PEDOT/PL electrode for the detection of 6 mM of hydroquinone in 100 mM of PBS buffer. The potential was swept from −0.5~0.7 V with different scan rate (B) The effect of temperature on the anodic peak current.
